# UCHL3 Regulates Topoisomerase-Induced Chromosomal Break Repair by Controlling TDP1 Proteostasis

**DOI:** 10.1016/j.celrep.2018.05.033

**Published:** 2018-06-13

**Authors:** Chunyan Liao, Ryan Beveridge, Jessica J.R. Hudson, Jacob D. Parker, Shih-Chieh Chiang, Swagat Ray, Mohamed E. Ashour, Ian Sudbery, Mark J. Dickman, Sherif F. El-Khamisy

**Affiliations:** 1 Krebs Institute, Department of Molecular Biology and Biotechnology, Firth Court, University of Sheffield, S10 2TN Sheffield, UK; 2 Genome Damage and Stability Centre, University of Sussex, Brighton, UK; 3 Center for Genomics, Helmy Institute, Zewail City of Science and Technology, Giza, Egypt; 4 Department of Chemical and Biological Engineering, University of Sheffield, Sheffield, UK

**Keywords:** TDP, topoisomerase, aging, neurodegeneration, cancer, rhabdosarcoma, SCAN1, heart failure, myocardial infarction, UCHL3

## Abstract

Genomic damage can feature DNA-protein crosslinks whereby their acute accumulation is utilized to treat cancer and progressive accumulation causes neurodegeneration. This is typified by tyrosyl DNA phosphodiesterase 1 (TDP1), which repairs topoisomerase-mediated chromosomal breaks. Although TDP1 levels vary in multiple clinical settings, the mechanism underpinning this variation is unknown. We reveal that TDP1 is controlled by ubiquitylation and identify UCHL3 as the deubiquitylase that controls TDP1 proteostasis. Depletion of UCHL3 increases TDP1 ubiquitylation and turnover rate and sensitizes cells to TOP1 poisons. Overexpression of UCHL3, but not a catalytically inactive mutant, suppresses TDP1 ubiquitylation and turnover rate. TDP1 overexpression in the topoisomerase therapy-resistant rhabdomyosarcoma is driven by UCHL3 overexpression. In contrast, UCHL3 is downregulated in spinocerebellar ataxia with axonal neuropathy (SCAN1), causing elevated levels of TDP1 ubiquitylation and faster turnover rate. These data establish UCHL3 as a regulator of TDP1 proteostasis and, consequently, a fine-tuner of protein-linked DNA break repair.

## Highlights

• TDP1 proteostasis is controlled by a UCHL3-dependent ubiquitylation mechanism
• UCHL3 depletion sensitizes mammalian cells to TOP1 inhibitors
• Increased TDP1 protein in rhabdomyosarcoma is driven by UCHL3 upregulation
• Decreased TDP1 protein in spinocerebellar ataxia is driven by UCHL3 downregulation

## Graphical Abstract

**Figure:**
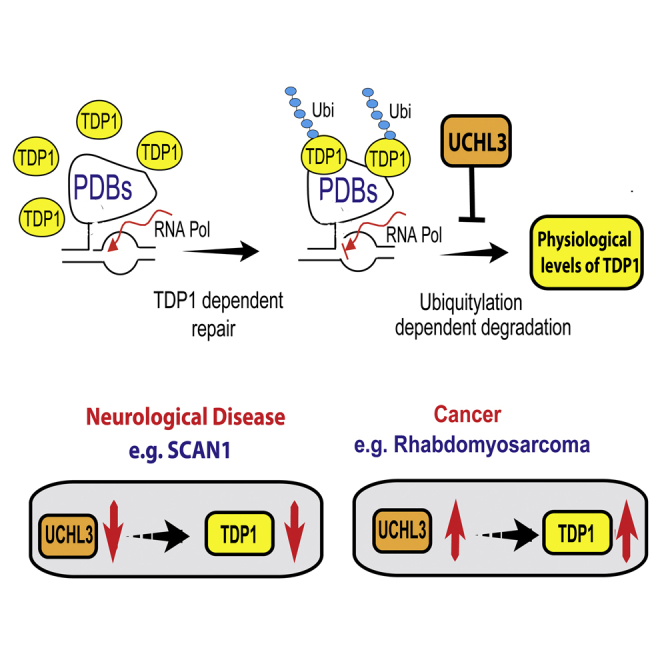

## Teaser

Deciphering the mechanisms regulating protein homeostasis is clinically important. Liao et al. identify UCHL3 as a key player in ubiquitylation and turnover of the DNA repair enzyme TDP1. Disruption of TDP1’s ubiquitylation status causes neurological disease or resistance to topoisomerase I-targeting chemotherapy in cancer.

## Introduction

Optimal protein homeostasis (proteostasis) is essential for all aspects of cellular activities. It is controlled by a number of competing, but integrated, pathways including biogenesis, trafficking, and degradation (Balch et al., 2008, Meerang et al., 2011). Adjusting these processes to the demand of individual proteins and pathways is essential to maintain cellular function. Perturbation in proteostasis causes human disease. For example, failure to degrade and subsequently clear proteins that are covalently linked to DNA cause human neurological disease and premature aging (El-Khamisy et al., 2005, Gómez-Herreros et al., 2014, Rubinsztein, 2006, Vaz et al., 2016, Walker et al., 2017). The linkage of proteins to DNA could be non-enzymatically driven by the vicinity of proteins to DNA in the presence of endogenous crosslinking agents, such as aldehydes. Formaldehyde is a potent crosslinking agent generated as a metabolic by-product during de-methylation of histones and DNA (Shi et al., 2004, Trewick et al., 2002). It could also be driven enzymatically, as part of physiological cycles of many DNA metabolising enzymes, such as topoisomerases, DNA glycosylases, and methyltransferases (Kiianitsa and Maizels, 2013). This linkage is generally transient and reversible, but it can become irreversible under certain physiological and pathological circumstances, causing deleterious protein-liked DNA breaks (PDBs). The most famous example of PDBs is those mediated by DNA topoisomerases (Ashour et al., 2015, Chiang et al., 2017, Pommier et al., 2016).

Topoisomerases are elegant biological tools that overcome topological entanglements inherent to the intertwined and compact nature of DNA. Their function is important for many aspects of DNA metabolism, such as gene transcription, DNA replication, recombination, and repair (Champoux and Dulbecco, 1972). Topoisomerases achieve this by transiently cleaving one or two strands of the DNA, thereby allowing the swiveling or rotation of the other strand or duplex around the break. Topoisomerase I (TOP1) generates intermediates in which the TOP1 is linked to the 3′ terminus of a single-strand break (SSB), whereas TOP2 intermediates are linked to the 5′ termini of a DNA double-strand break (DSB). Accumulation of TOP1- or TOP2-mediated PDBs cause neurological disease in humans (Katyal et al., 2014, Alagoz et al., 2013, El-Khamisy et al., 2005, Gómez-Herreros et al., 2014, Walker and El-Khamisy, 2018) and has been widely exploited in cancer chemotherapy (Alagoz et al., 2014, Ashour et al., 2015, Das et al., 2014, Meisenberg et al., 2017, Rehman et al., 2018). Accumulation of PDBs is counteracted by a number of PDB repair activities that constantly monitor and precisely disjoin the stalled topoisomerase from DNA termini or nucleolytically cut the DNA to release the stalled topoisomerase and a fragment of DNA. The former mode of repair spares the loss of genetic information and is conducted by a class of enzymes that specifically cleave the covalent linkage between the stalled topoisomerase and DNA called “tyrosyl DNA phosphodiesterases” (TDPs). TDP1 primarily disjoins stalled TOP1 peptides from PDBs, whereas TDP2 exhibits greater preference toward TOP2-mediated PDBs (El-Khamisy et al., 2005, Cortes Ledesma et al., 2009, Schellenberg et al., 2017). Defects in either TDP1 or TDP2 cause accumulation of PDBs and interfere with transcription, leading to neuronal cell death (Gómez-Herreros et al., 2014, Hudson et al., 2012, Katyal et al., 2007). In contrast, their overexpression has been linked to the resistance of cancer cells to topoisomerase targeting therapies (Barthelmes et al., 2004, Do et al., 2012, Duffy et al., 2016, Liu et al., 2007, Meisenberg et al., 2014). Despite their roles in many aspects of cellular activities and their implication in multiple clinical settings, the mechanisms that maintain TDPs proteostasis are not known.

TDP1 proteostasis is particularly attractive since a specific mutation in its active site that substitutes histidine 493 to arginine perturbs the completion of its catalytic cycle and additionally produces a TDP1-mediated PDB (Cuya et al., 2016, Interthal et al., 2005). Bi-allelic TDP1^H493R^ mutation is associated with ∼70% reduction of TDP1 protein level and leads to the accumulation of both TOP1- and TDP1-mediated PDBs, causing neurodegeration in spinocerebellar ataxia with axonal neuropathy 1 (SCAN1). Cells derived from SCAN1 patients exhibit marked sensitivity to TOP1 poisons, such as camptothecin and irinotecan (El-Khamisy et al., 2005, Interthal et al., 2005, Miao et al., 2006, Zhou et al., 2005). Although much is known about TDP1 biology, little is known about the mechanisms that control its steady-state level and why levels are markedly reduced in SCAN1 remains unexplained.

Post-translational modifications have been shown to control the steady-state level and modulate the function of several DNA repair factors (Elserafy and El-Khamisy, 2018, Meisenberg et al., 2012, Parsons et al., 2008). Protein ubiquitylation plays an important role in controlling the half-life of proteins via the ubiquitin-proteasome system (Amm et al., 2014). It offers a reversible mode of control for a myriad of cellular processes such as protein sorting, signal transduction and DNA repair (Chen and Chen, 2013, Francisco et al., 2014, Meisenberg et al., 2012, Parsons et al., 2008, Parsons et al., 2009, van Cuijk et al., 2014). The transient nature and fine tuning of ubiquitylation is achieved by an intricate balance of two opposing activities: the ubiquitin-conjugating cascade, which is a complex set of enzymes that conjugate ubiquitin to proteins, and deubiquitylase enzymes (DUBs), which remove ubiquitin molecules from the modified proteins (Komander et al., 2009, Metzger et al., 2014). Here, we report that TDP1 levels are regulated by ubiquitylation and identify UCHL3 as the deubiquitylase enzyme controlling TDP1 proteostasis. A low level of UCHL3 is associated with reduced TDP1 protein levels, causing neurological disease, whereas its overexpression causes higher TDP1 levels in cancer.

## Results

**Figure 1:**
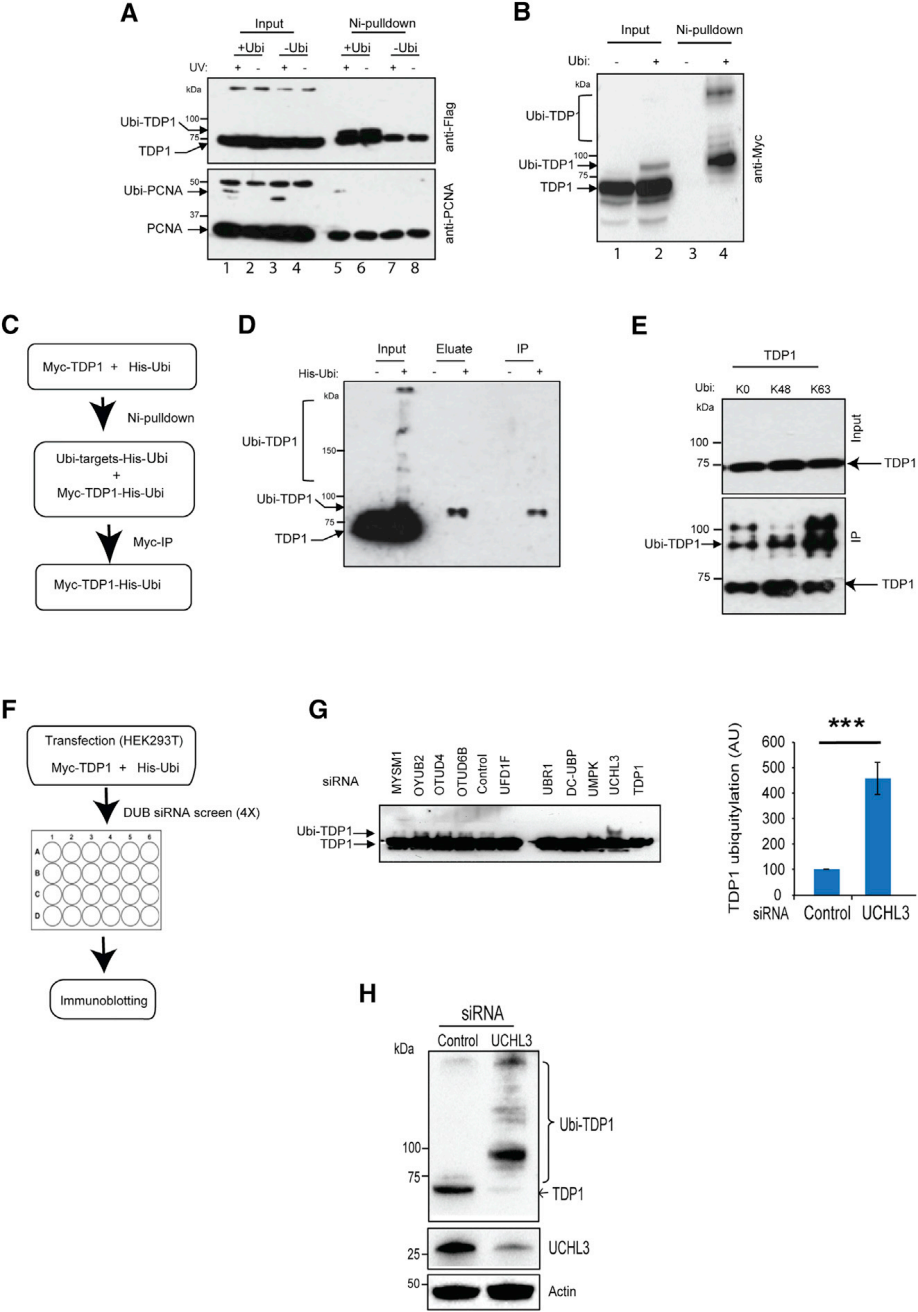
TDP1 Is Covalently Modified by Ubiquitin and Its Ubiquitylation Is Controlled by UCHL3 (A) HEK293T cells were transfected with plasmids encoding Flag-TDP1 and empty vector “−Ubi” or a vector encoding His-ubiquitin “+Ubi.” Cells were treated with 30J UV irradiation and allowed to recover for 1 hr. Ubiquitinated proteins were purified by Ni-pull-down and analyzed by immunoblotting. (B) HEK293T cells were transfected with plasmids encoding Myc-TDP1 and His-ubiquitin. Ubiquitinated proteins were purified by Ni-pull-down and analyzed by anti-Myc immunoblotting. (C) Diagram depicting purification of ubiquitinated TDP1 from mammalian cells. (D) HEK293T cells were transfected with plasmids encoding Myc-TDP1 and His-ubiquitin. Ubiquitinated proteins were purified by Ni-pull-down and Ni-bound proteins were eluted with imidazole “eluate” and subjected to immunoprecipitation with anti-Myc antibodies. (E) HEK293T cells were transfected with a plasmid encoding Myc-TDP1 and hemagglutinin (HA)-tagged wild-type ubiquitin “WT” or ubiquitin mutant in which all lysines were replaced by arginine “K0” or a mutant in which all lysines were replaced by arginine, except K48 “K48” or K63 “K63”. HA immunoprecipitates were purified using anti-HA antibodies and analyzed by anti-Myc immunoblotting. (F) A scheme depicting the siRNA DUB screen. (G) The DUB screen was repeated four times, and a representative immunoblot is shown (left). TDP1 ubiquitylation was quantified following normalization to TDP1 and is presented as average a.u. ± SEM (right). Asterisks denote p < 0.001; Student’s t test. (H) HEK293T cells were transfected with UCHL3 siRNA “UCHL3” or scrambled non-targeting siRNA “control,” and transfection was repeated after 48 hr, followed by cell lysis in SDS-denaturing buffer after another 24 hr. Endogenous TDP1 was analyzed by immunoblotting.

TDP1 overexpression in cancer is linked to resistance to topoisomerase I (TOP1)-targeting therapies (Duffy et al., 2016, Liu et al., 2007), and its reduced expression is linked to mutations that cause neurological disease (El-Khamisy et al., 2005, Takashima et al., 2002). To test whether TDP1 is a substrate for ubiquitylation, we transfected HEK293T cells with plasmids encoding Flag-TDP1 and empty vector or a vector encoding His-ubiquitin. Ubiquitinated proteins were purified by Ni-pull-down and fractionated on SDS-PAGE. As previously reported (Kannouche and Lehmann, 2004, Kannouche et al., 2004), a slower migrating band was observed for PCNA, a finding that is consistent with its ubiquitylation since it was dependent on UV irradiation (Figure 1A, lane 5). A slower migrating band was also observed for Flag-TDP1, which was only present in samples containing His-ubiquitin, suggesting that TDP1 is a substrate for ubiquitylation (Figure 1A, lanes 5 and 6). We noticed un-ubiquitylated Flag-TDP1 and PCNA coming down in the Ni-pull-down, which was not the case by repeating the experiment using Myc-TDP1 and more stringent pull-down conditions (Figure 1B). Next, we purified ubiquitinated proteins using Ni-pull-down, followed by elution with imidazole and the pool of ubiquitinated proteins was then subjected to immunoprecipitation using anti-Myc antibodies. This double purification method revealed a distinct band that was present in samples containing Myc-TDP1 and His-ubiquitin, but not Myc-TDP1 and empty His vector (Figures 1C and 1D), confirming that TDP1 is ubiquitinated in mammalian cells. Transfection with a ubiquitin mutant in which all lysine residues were replaced by arginine, except K48, led to less enrichement of monoubiquitinated TDP1 compared to ubiquitin mutants in which all lysines were replaced by arginine except K63 (Figure 1E), suggesting a role for ubiquitylation in controlling TDP1 proteostasis.

*In silico* analysis of TDP1 sequence using the ubiquitin site prediction tools UbPred, CKSAAP, and BDM-PUB revealed K186 and K425 as potential ubiquitylation sites. However, mutation of either or both residues to arginine did not abrogate TDP1 ubiquitylation (Figure S1A). Subjecting purified ubiquitinated TDP1 described in Figure 1D to mass spectrometric analysis using maXis HUR-TOF and a Q Exactive HF hybrid quadrupole orbitrap, in several other attempts, Thermo Orbitrap spectrometers, identified lysine 114 as a potential site. Mutant variants of TDP1 were generated at K114 and the nearby lysine residue K112 in addition to the known SUMOylation site K111, either separately or in combination (Figure S1B). However, none of the above attempts were successful, likely because of secondary ubiquitylation sites that can compensate if the primary site is lost. We therefore decided to study TDP1 ubiquitylation by identifying the deubiquitylase (DUB) activity. A small interfering RNA (siRNA) DUB screen was performed in which HEK293T cells were transfected with a plasmid encoding His-ubiquitin and Myc-TDP1 and then reverse transfected with an siRNA OnTarget Plus pooled library of all reported DUBs (Figure 1F). The DUB screen was repeated four times, and, doing so, revealed the ubiquitin carboxyl-terminal hydrolase isozyme L3 (UCHL3) as the most consistent hit (Figures 1G and S2). Prolonged depletion of UCHL3 using an independent pool of siRNA led to a marked reduction of endogenous TDP1 and a concomitant increase in slower-migrating bands, suggesting increased TDP1 ubiquitylation (Figure 1H).

**Figure 2:**
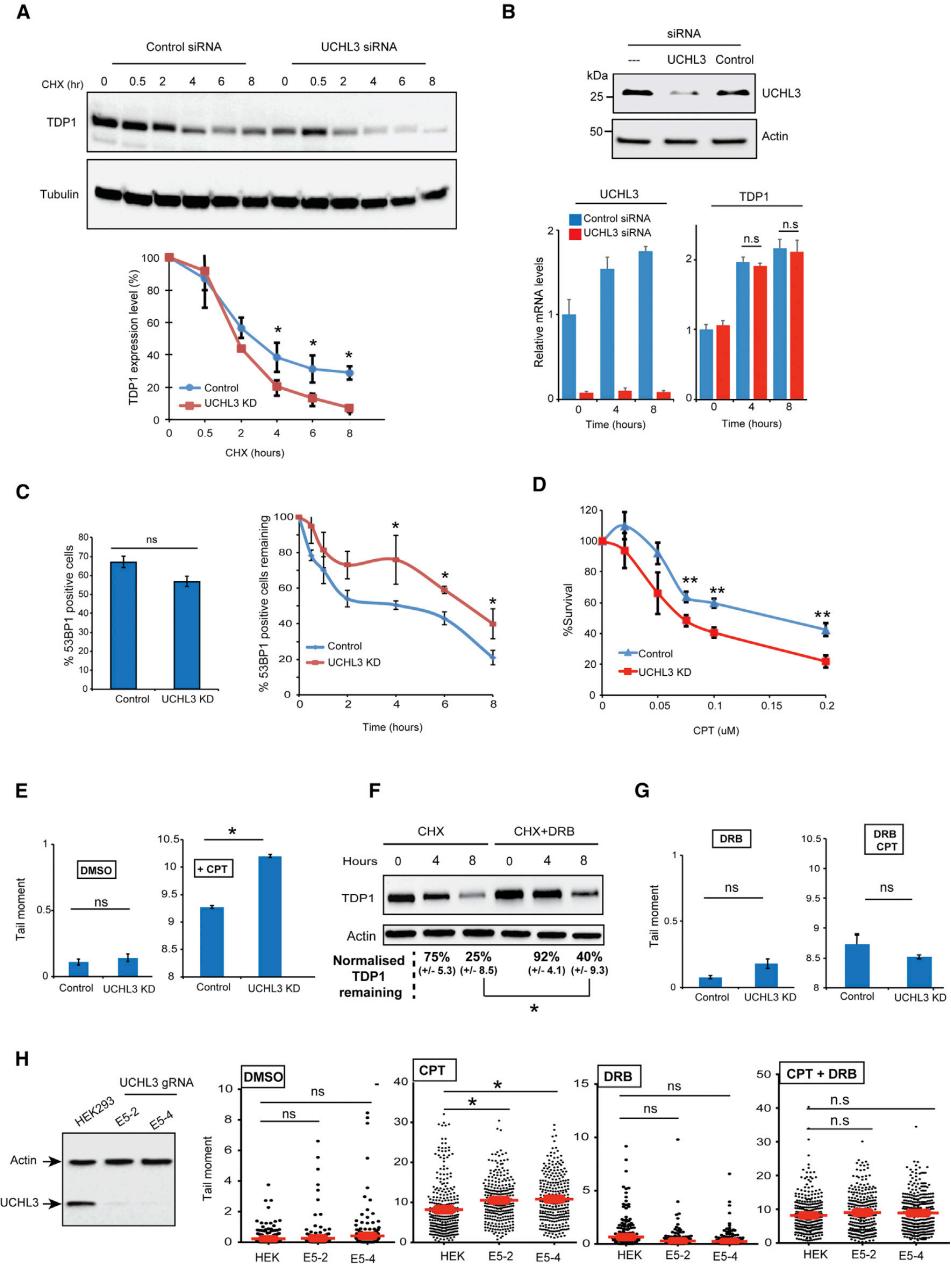
UCHL3 Is a Topoisomerase-Linked DNA Break Repair Factor (A) HEK293T cells were transfected with UCHL3 siRNA “UCHL3” or scrambled non-targeting siRNA “control,” followed by incubation with 100 μg/mL cycloheximide “CHX” for the indicated time periods. Endogenous levels of TDP1 were assessed by immunoblotting and quantified following normalization to tubulin and presented as an average a.u. ± SEM from three biological replicates. (B) HEK293T cells transfected with UCHL3 or non-targeting siRNA were analyzed by immunoblotting (top). TDP1 and UCHL3 mRNA were normalized to GAPDH from three biological replicates and presented as average ± SEM (bottom). (C) HEK293T cells transfected with UCHL3 siRNA “UCHL3” or scrambled non-targeting siRNA “control” were treated with 1 μM CPT for 30 min, and the number of cells positive for 53BP1 foci (containing more than 5 foci) were counted and presented as a percentage of total cells (left). The percentage of cells positive for 53BP1 was quantified at the indicted repair time points (right). Data are the average of three biological replicates ± SEM. (D) MRC5 cells were transfected with UCHL3 siRNA “UCHL3” or scrambled non-targeting siRNA “control” followed by incubation with the indicated concentrations of CPT for 1 hr, and survival was calculated from the average of three biological replicates ± SEM. (E) Chromosomal DNA breaks were quantified by alkaline comet assays, and data represent the average of three biological replicates ± SEM. 150 cells scored per experiment. (F) HEK293T cells expressing Myc-TDP1 were incubated with cycloheximide “CHX” alone or additionally with the transcription inhibitor, DRB, for the indicated time periods and Myc-TDP1 levels were assessed by immunoblotting. The remaining TDP1, following normalization to actin, was calculated from three biological replicates and presented as average ± SEM. (G) HEK293T cells were incubated with DMSO or 50 μM DRB, and chromosomal DNA breaks were quantified by alkaline comet assays. (H) HEK293T cells were transfected with CRSPR/Cas9 and gRNA targeting UCHL3, followed by isolation of two single clones and analyses of cell lysate by immunoblotting (left). CPT-induced DNA breaks were quantified using the alkaline comet assay as described in (E) and (G), and data are presented as scatterplots from three biological replicates. ^∗^p < 0.05; ^∗∗^p < 0.01; ^∗∗∗^p < 0.001; ns p > 0.05, Student’s t test.

To examine if the increased TDP1 ubiquitylation caused by UCHL3 depletion would lead to increased turnover, we monitored the TDP1 protein level following incubations with the protein synthesis inhibitor cycloheximide. UCHL3-depleted cells exhibited a faster rate of TDP1 turnover (Figure 2A), which was not due to an indirect impact on transcription, becuase UCHL3-deficient cells showed a reduction in UCHL3 mRNA, but not TDP1 mRNA (Figure 2B). While no difference in TOP1 double-strand breaks (DSBs) was observed immediately after CPT treatment, UCHL3-deficient cells exhibited a delay in the kinetics of TOP1-DSB clearance (Figure 2C). Furthermore, UCHL3-deficient cells were less able to survive the CPT challenge compared to controls, as measured by clonogenic survival assays (Figure 2D). Next, we quantified TOP1-mediated DNA strand breaks using the alkaline comet assay, which primarily measures DNA SSBs. Treatment with the TOP1 poison CPT led to elevation of TOP1-SSBs in UCHL3-deficient cells compared to controls (Figure 2E). Consistent with a predominant role of TDP1 during transcription (El-Khamisy et al., 2005), inhibition of transcription using 5,6-dichloro-1-β-D-ribofuranosylbenzimidazole (DRB) suppressed the turnover rate of TDP1 (Figure 2F) and abrogated the UCHL3-dependent difference in TOP1-SSBs (Figure 2G). Disrupting *UCHL3* using UCHL3 gRNA and CRISPR/Cas9 also led to higher accumulation of CPT-induced TOP1-SSBs, and the difference was also associated with active transcription, because it disappeared upon pre-incubation with DRB (Figure 2H). Together, these data suggest that UCHL3 is a player during TOP1-mediated DNA repair.

**Figure 3:**
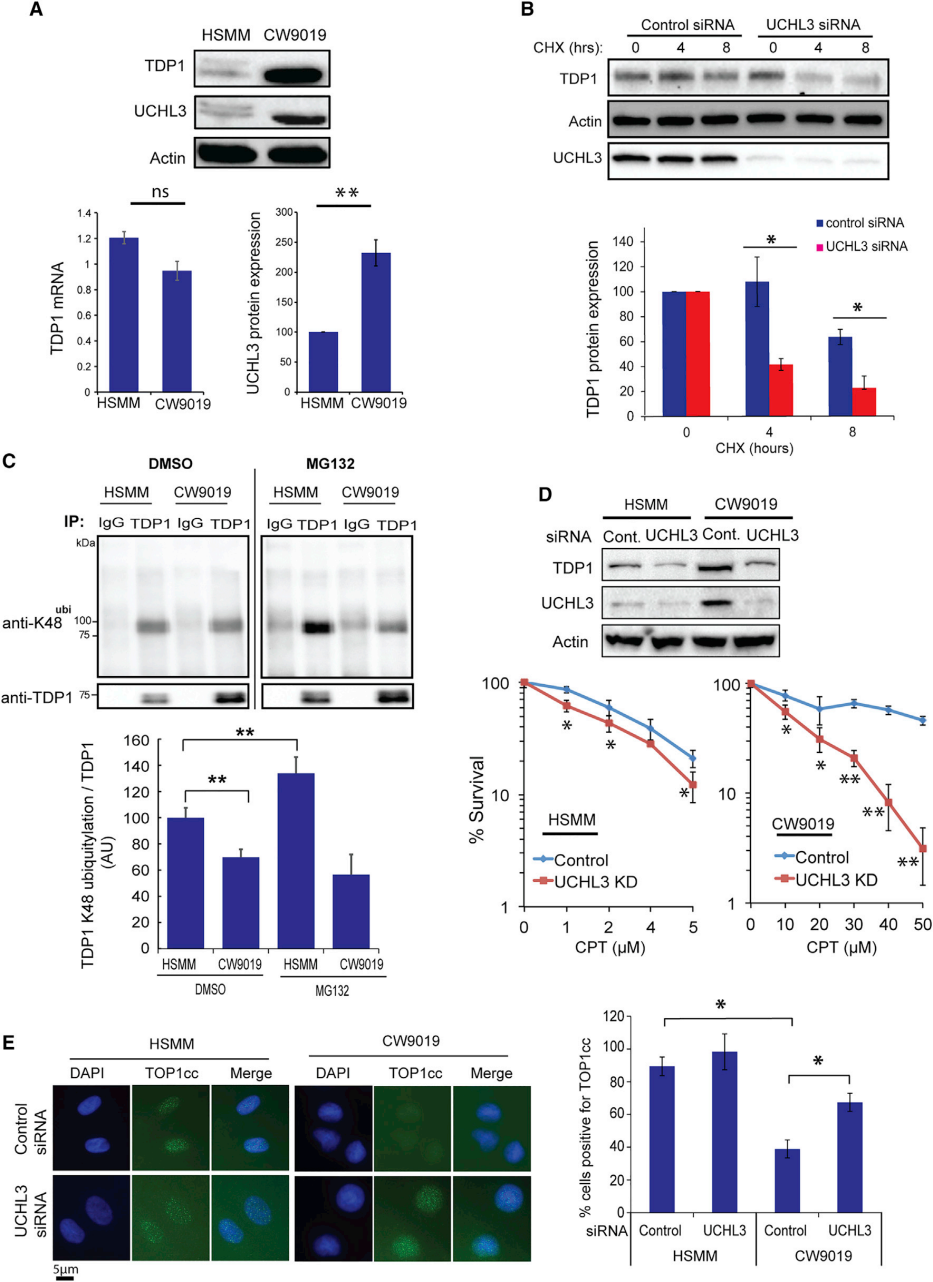
UCHL3 Is Upregulated in the TDP1-Overexpressing Rhabdomyosarcoma Cells and Its Depletion Restores TDP1 Levels (A) Control “HSMM” and rhabdomyosarcoma “CW9019” cell lysates analyzed by immunoblotting for TDP1 and UCHL3 expression. TDP1 mRNA was quantified by qPCR and is presented as an average fold change from three biological replicates ± SEM. UCHL3 protein levels were quantified from three biological repeats and are presented as average ± SEM. (B) CW9019 cells were transfected with scrambled non-targeting control or UCHL3 siRNA and, 48 hr later, were incubated with cycloheximide “CHX.” TDP1 levels were assessed by immunoblotting. Band intensities were quantified and normalized to actin. The remaining TDP1 was calculated relative to levels prior to CHX incubations from three biological replicates and is presented as average ± SEM. (C) HSMM and CW9019 cells were pre-incubated with 20 μM MG132 for 4 hr, followed by lysis and purification of TDP1 using anti-TDP1 immunoprecipitation. TDP1 immunoprecipitates were analyzed by anti-TDP1 and anti-K48 immunoblotting. TDP1 K48 ubiquitylation and TDP1 levels were quantified and presented as a percentage change of K48/TDP1 ratio. (D) Cells were transfected with scrambled non-targeting siRNA “control” or UCHL3 siRNA, and transfection was repeated after 48 hr. Cell lysates were collected 24 hr, following the last transfection and analyzed by immunoblotting (top). CW9019 cells were treated with the indicated doses of CPT and survival calculated using clonogenic survival assays (bottom). Results are averages of three repeats ± SEM. (E) Cells were transfected with scrambled non-targeting siRNA or UCHL3 siRNA and incubated with 1 μM CPT for 10 min and examined for TOP1cc using anti-TOP1cc antibodies. Scale bar, 5 μm. Percentage of positive cells with >8 TOP1cc foci were counted from three biological replicates and presented as an average ± SEM. ^∗^p < 0.05; ^∗∗^p < 0.01; ^∗∗∗^p < 0.001; ns p > 0.05, Student’s t test.

**Figure 4:**
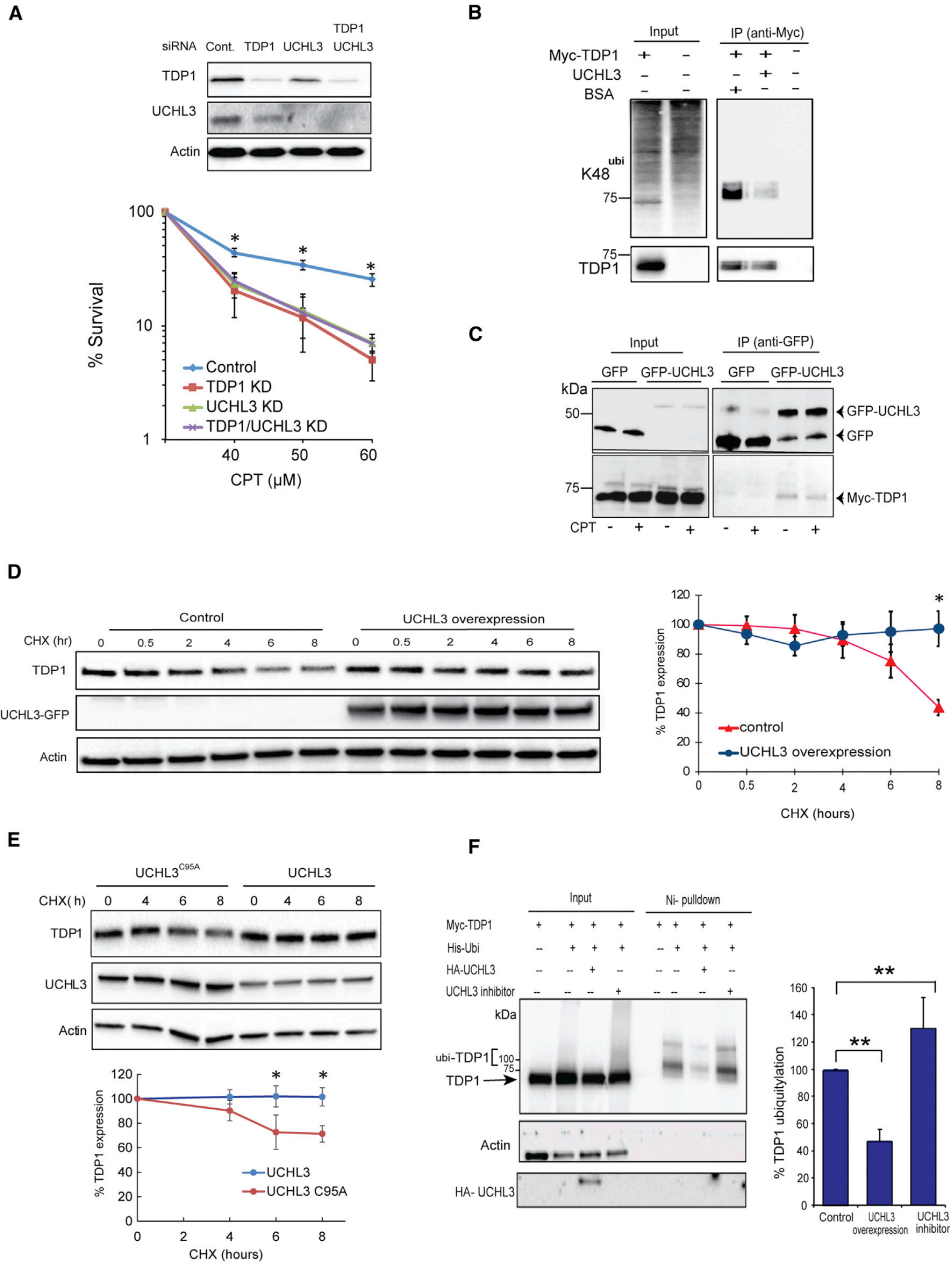
UCHL3 Acts Together with TDP1 to Regulate Topoisomerase-I-Mediated Chromosomal Break Repair (A) CW9019 cells were transfected with control non-targeting siRNA, TDP1 siRNA, UCHL3 siRNA, or both TDP1 and UCHL3 siRNA, and cell extracts were analyzed by immunoblotting (top). Cells transfected with the indicated siRNA were treated with CPT, and survival was calculated using clonogenic survival assays. Results are averages of four biological repeats ± SEM. (B) HEK293T cells expressing His-ubiquitin only or additionally Myc-TDP1 were treated with 20 μM MG132 for 4 hr, followed by lysis and purification of Myc-tagged proteins using Myc-trap beads. Purified Myc-TDP1 was incubated with BSA or recombinant UCHL3 for 4 hr at 30°C. Samples were analyzed by anti-Myc and anti-K48 immunoblotting. (C) HEK293T cells were transfected with Myc-TDP1 and either empty GFP or a vector encoding UCHL3-GFP. Cells were treated with DMSO or 10 μM CPT for 20 min, followed by lysis and purification of GFP-tagged proteins using GFP-trap beads, and then analyzed by anti-Myc and anti-GFP immunoblotting. (D) HEK293T cells were transfected with an empty vector “control” or plasmid encoding UCHL3-GFP, followed by incubation with 100 μg/mL cycloheximide “CHX” for the indicated time periods. Endogenous TDP1 were assessed by immunoblotting, normalized to actin, and the remaining TDP1 level was calculated relative to levels prior to CHX incubations from three biological replicates and presented as average ± SEM. (E) TDP1 levels were quantified from HEK293T cell lysates containing wild-type UCHL3 or the catalytically inactive UCHL3^C95A^ mutant following incubation with 100 μg/mL CHX for the indicated time periods. Data represent the average of three biological replicates ± SEM. (F) HEK293T cells containing Myc-TDP1 and His-ubiquitin were incubated with the UCHL3 inhibitor 1,3-indanedione (30675-13-9, Santa Cruz). Levels of ubiquitinated proteins were purified by Ni-pull-down and analyzed by anti-Myc, anti-HA, and anti-actin antibodies. TDP1 ubiquitylation was quantified and is presented as a percentage change relative to control cells transfected with Myc-TDP1 and His-ubiquitin from three biological replicates. ^∗^p < 0.05; ^∗∗^p < 0.01; ^∗∗∗^p < 0.001; ns, p > 0.05; Student’s t test.

The acute inhibition of TOP1-mediated DNA repair has been widely exploited to treat various forms of solid tumors (Ashour et al., 2015, Pommier et al., 2016). However, cancer cells can resist TOP1 targeting therapies by overexpression of PDB repair factors such as TDP1. For example, recent elegant work identified TDP1 overexpression in rhabdomyosarcoma (Duffy et al., 2016). Consistent with published data, TDP1 is markedly overexpressed in the CW9019 rhabdomyosarcoma compared to control cells (Figure 3A, top). Interestingly, the overexpression was not due to elevated TDP1 mRNA, but, instead, we observed ∼2.5-fold higher UCHL3 protein expression (Figure 3A, bottom). Depletion of UCHL3 in rhabdomyosarcoma cells markedly accelerated TDP1 turnover, as measured by CHX chase experiments (Figure 3B). In line with UCHL3 overexpression, immunoprecipitation of endogenous TDP1 revealed lower levels of K48-ubiquitylated TDP1 in rhabdomyosarcoma compared to control cells (Figure 3C). Inhibiting the proteasome using MG132 led to marked enrichment of K48-ubiquitylated TDP1 in control cells but no impact in rhabdomyosarcoma cells, due to high UCHL3 levels (Figure 3C). Depletion of UCHL3 was sufficient to reduce TDP1 expression in rhabdomyosarcoma cells to levels comparable to control cells (Figure 3D, top) and led to hypersensitivity to the TOP1 poison CPT (Figure 3D, bottom). Consistent with the higher level of UCHL3 in rhabdomyosarcoma, they exhibited lower levels of CPT-induced topoisomerase cleavage complexes (TOP1cc), which were markedly increased upon UCHL3 depletion (Figure 3E). To ascertain a direct role of UCHL3 during TDP1-mediated repair, we performed epistasis analyses. Co-depletion of TDP1 and UCHL3 did not lead to more CPT hypersensitivity compared to depletion of either enzyme alone confirming that they both act together in the same pathway to repair TOP1-mediated PDBs (Figure 4A). Furthermore, incubation of purified K48-ubiquitylated TDP1 with recombinant UCHL3 led to reduction of TDP1 ubiquitylation (Figure 4B) and a physical interaction between TDP1 and UCHL3 was observed by co-immunoprecipitation (Figure 4C). Ectopic overexpression of UCHL3 led to stabilization of TDP1 protein level (Figure 4D), which was not the case for a UCHL3^C95A^ catalytically inactive mutant (Zhang et al., 2013) (Figure 4E). Furthermore, catalytic inhibition of UCHL3 led to increased TDP1 ubiquitylation (Figure 4F). Together, we conclude that UCHL3 controls PDB repair by regulating TDP1 proteostasis.

**Figure 5:**
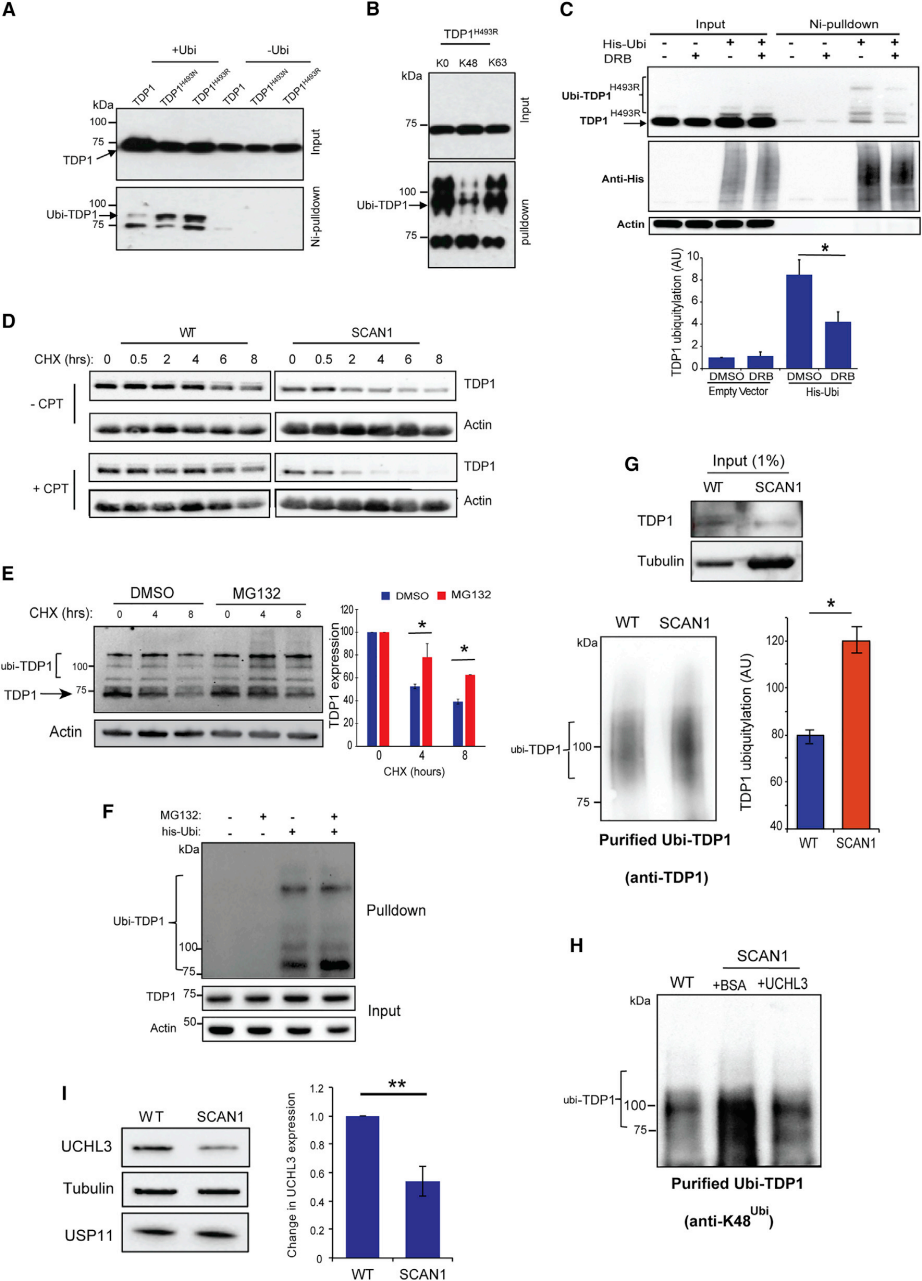
TDP1 in Patient-Derived SCAN1 Cells Exhibits Elevated Ubiquitylation and Faster Turnover Rate (A) HEK293T cells were transfected with plasmids encoding His-ubiquitin and Myc-TDP1, Myc-TDP1^H493N^, or Myc-TDP1^H493R^. Ubiquitinated proteins were purified by Ni-pull-down and analyzed by anti-Myc immunoblotting. (B) HEK293T cells were transfected with a plasmid encoding Myc-TDP1^H493R^ and HA-tagged wild-type ubiquitin “WT” or ubiquitin mutant in which all lysines were replaced by arginine “K0” or a mutant in which all lysines were replaced by arginine, except K48 “K48” or K63 “K63”. HA immunoprecipitates were purified using anti-HA antibodies and analyzed by anti-Myc immunoblotting. (C) HEK293T cells were transfected with a plasmid encoding Myc-TDP1 ^H493R^ and empty vector “−” or plasmids encoding His-ubiquitin “His-Ubi.” Cells were treated with 50 μM DRB for 2 hr, and ubiquitinated proteins were purified by Ni-pull-down and analyzed by immunoblotting. TDP1 ubiquitylation was quantified following normalization to TDP1 and is presented as an average a.u. ± SEM from three biological replicates. (D) Control “WT” or SCAN1 human lymphoblastoid cells were incubated with 100 μg/mL CHX and mock treated with either DMSO “−CPT” or 10 μM CPT “+CPT” for the indicated time periods. Endogenous levels of TDP1 were assessed by immunoblotting. (E) SCAN1 cells (2.5 × 10^6^) were incubated with 100 μg/mL CHX and mock treated with either DMSO or 20 μM MG132 for the indicated time periods. Endogenous TDP1 was assessed by immunoblotting. Data represent the average of three biological replicates ± SEM. (F) HEK293T cells expressing Myc-TDP1^H493R^ and His-ubiquitin were treated with 20 μM MG132 or DMSO for 4 hr. The ubiquitinated proteins were enriched using Ni-pull-down, and ubiquitinated TDP1 was analyzed by anti-Myc immunoblotting. (G) Control (2 × 10^7^) “WT” human lymphoblastoid cells or 2 × 10^8^ SCAN1 cells were lysed in ubiquitin binding buffer. 1 mg of WT lysate or 10 mg SCAN1 lysate was incubated with 0.3 μL or 3 μL of the ubiquitin binding resin Multi-DSK, respectively. Multi-DSK-bound ubiquitinated proteins were purified by 300 mM imidazole elution, and ubiquitinated proteins were subjected to TDP1 immunoprecipitation and ubiquitinated TDP1 analyzed by anti-TDP1 immunoblotting. TDP1 ubiquitylation was quantified, normalized to TDP1, and presented as an average a.u. ± SEM. Data are the average of three biological replicates. (H) WT and SCAN1 cells were treated as described in (G). Lysates were incubated with Multi-DSK, and bound ubiquitinated proteins were subjected to TDP1 immunoprecipitation. Purified ubiquitylated TDP1 was incubated with BSA or recombinant UCHL3 for 4 hr at 30°C and analyzed by anti-K48 immunoblotting. (I) Lysates from control “WT” and SCAN1 cells were analyzed by immunoblotting. UCHL3 expression was normalized to tubulin and presented as a percentage change in SCAN1 cells relative to control WT cells. Data are the average of three biological replicates ± SEM. ^∗^p < 0.05; ^∗∗^p < 0.01; ^∗∗∗^p < 0.001; ns, p > 0.05; Student’s t test.

In contrast to CPT-resistant cancers, progressive accumulation of PDBs due to TDP1 deficiency leads to neurodegeneration. Mutation of histidine 493 of TDP1 to arginine causes spinocerebellar ataxia and axonal neuropathy 1 (SCAN1) (Takashima et al., 2002). Cells derived from SCAN1 patients exhibit a marked elevation of PDBs and a significant reduction of TDP1 protein level (El-Khamisy et al., 2005, Interthal et al., 2005). However, the reason for this reduction remains unknown. We therefore wondered whether the TDP1 H493 mutation would be more prone to ubiquitylation than wild-type TDP1. To test this, we performed Ni-pull-down comparing TDP1, TDP1^H493R^ and another mutant that has been shown to inhibit the second step of TDP1 catalytic cycle, TDP1^H493N^ (Interthal et al., 2005). Mutations in H493 that lead to accumulation of PDBs do indeed render TDP1 more prone to ubiquitylation (Figure 5A). Mutating all lysines in ubiquitin to arginine except K48 led to reduced enrichement of monoubiquitinated TDP1^H493R^ (Figure 5B). Consistent with the accumulation of TDP1^H493R^ PDBs during transcription, incubation with the transcription inhibitor, DRB, led to a reduction of TDP1^H493R^ ubiquitylation (Figure 5C). These data suggest that accumulation of unrepaired PDBs, including DNA-TDP1^H493R^ breaks during transcription, promotes TDP1^H493R^ ubiquitylation. To test whether this is true in patient-derived SCAN1 cells, we monitored TDP1 protein turnover following treatment with cycloheximide. TDP1^H493R^ in SCAN1 cells exhibited faster rate of degradation compared to wild-type TDP1 (Figure 5D). Treatment with CPT led to stabilization of TDP1 in wild-type cells, which is consistent with previous reports (Das et al., 2009); however, it led to a faster rate of degradation in SCAN1 cells (Figure 5D). Inhibiting the proteasome using MG132 suppressed the fast rate of TDP1^H493R^ degradation and led to accumulation of ubiquitylated TDP1^H493R^ species (Figures 5E and 5F). Purification of endogenous ubiquitylated TDP1 using Multi-DSK (Wilson et al., 2012) followed by TDP1 immunoprecipitation using anti-TDP1 antibodies revealed a marked increase of endogenous TDP1 ubiquitylation in SCAN1 cells (Figure 5G). The ubiquitylation of purified K48-ubiquitylated TDP1 from SCAN1 cells was reduced upon treatment with recombinant UCHL3 (Figure 5H). Consistent with this, SCAN1 cells exhibited ∼50% reduction of UCHL3 expression (Figure 5I).

**Figure 6:**
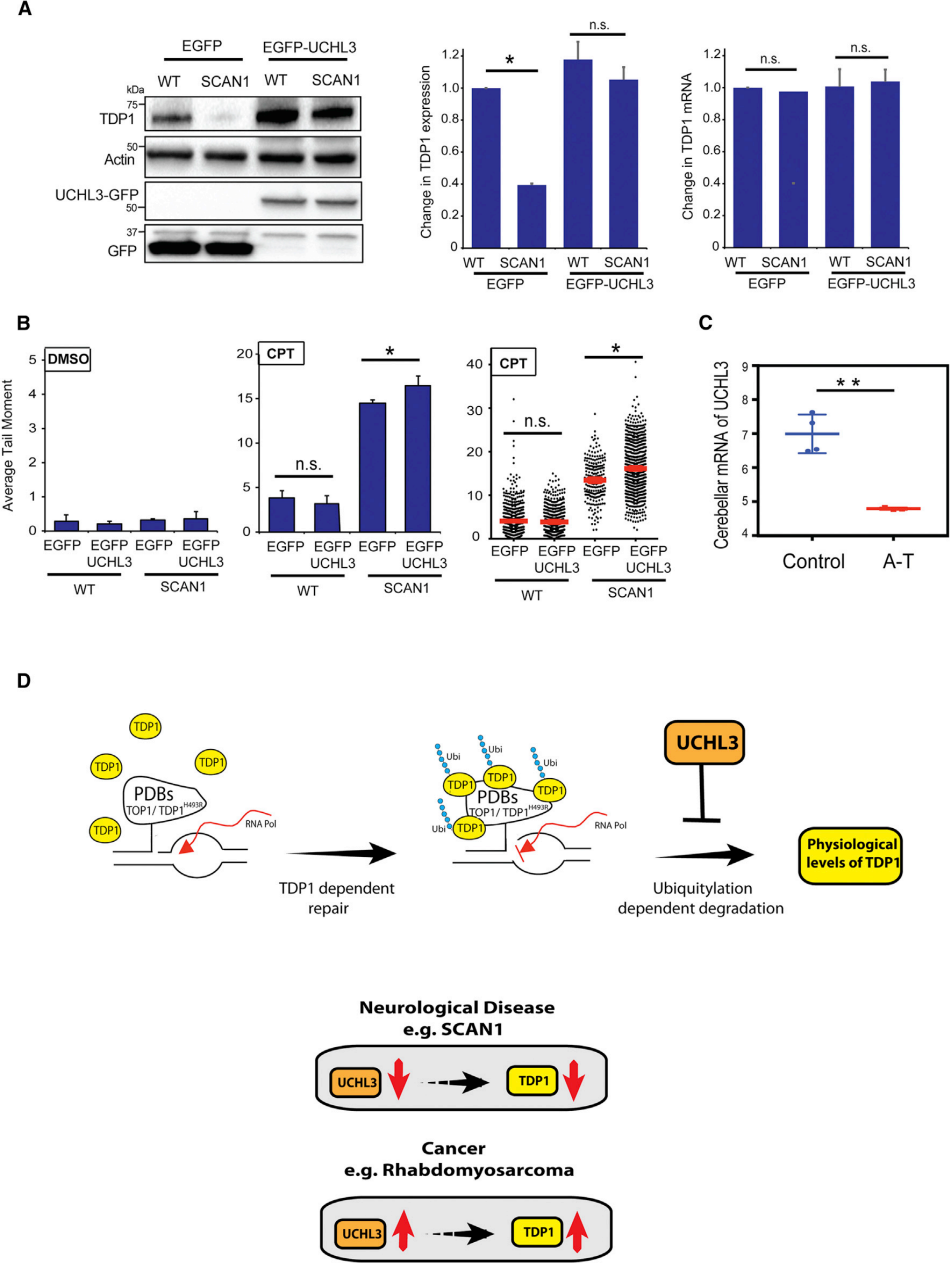
UCHL3 Overexpression Restores the Level of TDP1 in Patient-Derived SCAN1 Cells (A) Control wild-type “WT” and SCAN1 cells were transfected with empty EGFP plasmid or EGFP-UCHL3 plasmid encoding full-length UCHL3. Cell lysates were analyzed by immunoblotting using anti-UCHL3, anti-TDP1, and anti-GFP antibodies (left). TDP1 protein expression level was quantified from three biological repeats and presented as average relative to levels in control cells ± SEM (middle). Total RNA was extracted from cells described in (A), followed by quantification of TDP1 mRNA using qPCR. Following normalization to GAPDH, the fold change in mRNA in SCAN1 cells relative to WT cells is presented from three biological replicates ± SEM (right). (B) CPT-induced DNA strand breaks were quantified by alkaline comet assays, and data represent the average of three biological replicates ± SEM. 150 cells scored per experiment (left and middle). Scatterplot showing individual tail moments of the three replicates from cells treated with CPT (right). (C) Microarray data from wild-type control and ataxia Telangiectasia A-T patient cerebella were normalized, and a differential expression analysis was performed in R studio and presented as log2 normalized mRNA levels. (D) A model depicting the role of UCHL3 during topoisomerase-mediated repair. PDBs can be formed by stalling of TOP1 or by specific mutations in enzymes involved in PDB repair, such as TDP1^H493R^. The persistence of PDBs during transcription interferes with RNA polymerases “RNA Pol” and triggers the ubiquitylation of TDP1, channeling it to proteasomal degradation. The degradation is kept under control through the deubiquitylase activity of UCHL3. If UCHL3 levels go down, like in SCAN1, the TDP1 protein level goes down. In contrast, if UCHL3 goes up, like in rhabdomyosarcoma, the TDP1 level goes up. The inappropriate level of UCHL3 perturbs the homeostasis of PDB repair, causing neurological disease in SCAN1 or cancer resistance to TOP1-targeting therapies in rhabdomyosarcoma. ^∗^p < 0.05; ^∗∗^p < 0.01; ^∗∗∗^p < 0.001; ns, p > 0.05; Student’s t test.

Replenishing UCHL3 levels in SCAN1 by its ectopic overexpression was sufficient to restore TDP1 protein, without affecting TDP1 mRNA, to levels comparable to control cells (Figure 6A). Although it may appear beneficial to restore the reduced level of TDP1 in SCAN1, this variant carries the bi-allelic TDP1^H493R^ mutation, which generates PDBs featuring both TOP1- and TDP1-linked DNA breaks. Consistent with this, overexpression of UCHL3 led to a further increase in PDBs in SCAN1 cells, as measured by the alkaline comet assay (Figure 6B). It appears therefore that SCAN1 cells downregulate UCHL3 in an attempt to avoid the unfavorable outcomes of accumulating further PDBs. Finally, the accumulation of PDBs is also a feature of other neurological disorders with defective ATM, such as ataxia telangiectasia (A-T) and amyotrophic lateral sclerosis (ALS) (Alagoz et al., 2013, Katyal et al., 2014, Walker et al., 2017). To test whether UCHL3 is also downregulated in A-T, we compared microarray data (GEO: GSE61019) obtained from the cerebellum of control apparently healthy individuals to those obtained from A-T patients (Jiang et al., 2015). Consistent with the SCAN1 patient-derived data, UCHL3 is also downregulated in the human A-T cerebellum. Thus, we conclude that UCHL3 is a player in PDB repair and its level is downregulated in at least two PDB repair-deficient human neurological disorders.

## Discussion

TOP1-mediated PDBs are an important class of DNA single-strand breaks (SSBs), which are widely exploited in chemotherapy (Ashour et al., 2015, Pommier et al., 2016). In contrast, their progressive accumulation leads to multiple neurological disorders (Alagoz et al., 2013, El-Khamisy, 2011, Gómez-Herreros et al., 2014, Hudson et al., 2012, Katyal et al., 2014, Kerzendorfer et al., 2010, Walker et al., 2017). TDP1 is a key factor for repairing TOP1-mediated PDBs and its mutation causes the demise of post-mitotic tissue and cerebellar ataxia in SCAN1. Although much is known about the enzymatic and cellular functions of TDP1, it is not known how cells fine-tune its level to support PDB repair events. Here, we report multiple evidence showing a direct role of UCHL3 in controlling TDP1 proteostasis: (1) reduction of K48-ubiquitylated TDP1 in cancer cells overexpressing UCHL3, (2) reduction of K48-uibiquitylated TDP1 upon addition of recombinant UCHL3, (3) enrichment of K48-ubiquitylated TDP1 upon MG132 treatment, which is dependent on UCHL3 levels, (4) epistatic relationship showing that co-depletion of TDP1 and UCHL3 does not further sensitize cells to TOP1 poisons than depletion of either enzyme alone, (5) physical interaction between TDP1 and UCHL3, and (6) changes in the ubiquitylation status and TDP1 turnover upon changes in UCHL3 levels induced by its ectopic overexpression, siRNA depletion, CRISPR editing, and pharmacologic inhibition and, also, in two physiologically relevant clinical settings.

Like TOP1 and TDP1, UCHL3 homologs have been identified across multiple species, suggesting the evolutionarily conserved nature of this mode of DNA damage repair regulation (Frickel et al., 2007). Protein ubiquitylation has been shown to play a key role during DNA damage repair (Jackson and Durocher, 2013). For example, histone ubiquitylation promotes the recruitment of DSB repair factors to sites of chromatin damage (Huen et al., 2007, Doil et al., 2009, Mailand et al., 2007, Mattiroli et al., 2012), and regulates the level of base excision repair proteins (Meisenberg et al., 2012, Parsons et al., 2008, Parsons et al., 2009). Here, we identify UCHL3 as a regulator for chromosomal SSB repair by controlling the ubiquitylation status and therefore the steady-state level of TDP1. UCHL3 is a cysteine protease featuring a classical Cys-His-Asp catalytic triad. There is a precedent for the involvement of UCHL3 in controlling steady-state levels of proteins in other cellular processes. For example, UCHL3 physically interacts with Smad1 and decreases its poly-ubiquitylation (Kim et al., 2011). Consequently, it was suggested that UCHL3 stabilizes Smad1 signaling, which may explain the enhanced osteoblast differentiation caused by UCHL3 overexpression (Kim et al., 2011). Deletion of *Uchl3* in mice led to marked accumulation of poly-ubiquitinated proteins (Setsuie et al., 2010). This accumulation was due to UCHL3 catalytic activity since accumulation of poly-ubiquitinated proteins in *Uchl3−/−* MEFs was attenuated by the exogenous expression of wild-type, but not a hydrolase-deficient mutant (Setsuie et al., 2010). UCHL3 has been reported to deubiquitylate RAD51 and facilitates DSB repair by homologous recombination (Luo et al., 2016).

This study provides an answer to the long-standing question about why TDP1 levels in SCAN1 are reduced by ∼70%. We reveal that the TDP1 mutation in SCAN1 predisposes the enzyme to higher ubiquitylation levels, due to the elevated formation of transcription-blocking PDBs, which consequently accelerates its proteasomal degradation (Figure 6D). Suppressing the increased TDP1 ubiquitylation in SCAN1 cells by overexpression of UCHL3 was sufficient to restore TDP1 protein to levels comparable to control cells. These findings unravel the importance of UCHL3 to regulate chromosomal repair events that are particularly important for the maintenance of the nervous system. Notably, UCHL3 is expressed in the brain and its deletion in mice has been reported to cause neurodegeneration (Setsuie et al., 2009), retinal degeneration (Sano et al., 2006), and significant memory impairment (Wood et al., 2005). Furthermore, using the senescence-accelerated mouse prone 8 (SAMP8) as a model to study cognitive decline during aging, it has been reported that UCHL3 is markedly downregulated in the hippocampus; a critical brain region associated with cognitive decline during aging and various neurodegenerative disorders (Wang et al., 2008). These observations are particularly attractive and consistent with our findings, since downregulation of UCHL3 causes marked reduction in TDP1 levels and, consequently, compromises the repair of transcription blocking PDBs, the accumulation of which has been linked to neurodegeration and premature aging (Garinis et al., 2009). It is therefore plausible to propose that PDBs are elevated in the hippocampus of SAMP8 mice, providing a mechanistic explanation for the neuronal demise associated with aging in this model. At the biochemical level, the prominent accumulation of monoubiquitinated TDP1 in UCHL3 depleted cells suggests a preference for UCHL3 in clipping off the entire ubiquitin chain instead of processive cleavage, which remains to be tested in cell-free assays.

From the cancer point of view, UCHL3 is an attractive “druggable” target and previous efforts focused on developing UCHL3 inhibitors that specifically target UCHL3 in plasmodium falciparum parasite, offering promising selectivity due to structural differences compared to human UCHL3 (Artavanis-Tsakonas et al., 2010). Here, we report that cancer cells overexpressing TDP1, such as rhabdomyosarcoma, are associated with a concomitant overexpression of UCHL3. Depletion of UCHL3 in rhabdomyosarcoma cells reduced TDP1 levels and sensitized cells to TOP1 poisons. Consistent with our data, UCHL3 overexpression in breast cancer has been correlated with poor survival rates (Luo et al., 2016). Thus, UCHL3 inhibition may offer a worthy therapeutic opportunity for cancer cells that possess upregulated PDB repair mechanisms, particularly those that exploit this upregulation as a mechanism to resist TOP1 targeting chemotherapy.

In summary, this study identifies UCHL3 as a regulator of chromosomal SSB repair by controlling TDP1 proteostasis and highlights it physiological significance in neurological disease and cancer.

## Experimental Procedures

### Immunoprecipitation

HEK293T cells were plated at 1 × 10^6^ per 10-cm dish and transfected using standard calcium phosphate precipitation. Cells were lysed in buffer containing 50 mM Tris (pH 8), 0.5% Triton X, 40 mM NaCl, 2 mM CaCl_2_, 20 mM nethylmaleimide, 1× protease inhibitor (Roche), 1× phosphatase inhibitor (Roche), and 25 U/mL Basemuncher (Expedeon). Lysates were kept on ice for 20 min before clearing by centrifugation at 13,500 rpm at 4°C for 15 min. Input samples were kept at −20°C. Lysates were then adjusted to 150 mM NaCl; anti-Myc antibody was added (final concentration ∼2 μg/mL); and samples were left rotating at 4°C for 1 hr. Protein G beads or GFP-Trap beads (chromotek) were washed three times in wash buffer (20 mM Tris [pH 8], 150 mM NaCl, and 20 mM nethylmaleimide) before the addition of lysate. Samples were then rotated overnight at 4°C. Beads were washed three times with wash buffer before resuspension in SDS loading buffer for analysis by immunoblotting. If Ni-NTA (Expedeon) beads were used for immunoprecipitation, lysates were adjusted to 150 mM NaCl and 30 mM imidazole. Wash buffer was also adjusted to 30 mM imidazole, while wash buffer containing 300 mM imidazole was used to elute bound proteins.

### DUB Screen

A Human ON-TARGET plus siRNA library for all known DUBs (G-104705-05, GE Life Sciences) was replated from 96- to 24-well formats and stored at −80°C. 24-well plates were designed so that each would have a non-targeting control and TDP1 (transfection control) siRNA well. HEK293T cells were plated at 1 × 10^6^ per 10-cm dish and DNA transfected using calcium phosphate precipitation. 24 hrs after DNA transfection, siRNA library plates (24 well) were defrosted, and 100 μL of MEM (not supplemented) containing 0.5 μL Dharmafect 1 (GE Life Sciences) was added to each well and incubated for 20 min. DNA-transfected cells were trypsinised, replated onto 24-well plates at a density of 2.2 × 10^5^/well, and incubated for a further 48 hr. Cells were washed twice with 0.5 mL PBS; all PBS was removed before the addition of 40 μL SDS loading buffer; and cells were vortexed 3 times for ∼15 s. Lysates were then boiled for 10 min and clarified by centrifugation at 10,000 × *g* for 1 min. Samples were then analyzed by immunoblotting using an 8% gel and anti-TDP1 antibody. Validation of putative hits was conducted by seeding HEK293T cells in 6-well plates, followed by transfection of separate siRNA sequences and 4 pooled siRNA sequences against the hit, 24 hr later, using DharmaFECT 1 Transfection Reagent (GE Dharmacon,T-2001-01). Cells were lysed with SDS-PAGE and analyzed by immunoblotting.

### Purification of Endogenous Ubiquitylated Proteins and *In Vitro* Deubiquitination Assays

Cells were lysed in a buffer containing 50 mM Tris, 150 mM NaCl, 1.5 mM MgCl_2_, 5 mM EDTA, 10% Glycerol, 1% Triton, 1× phosphatase inhibitors, 1× protease inhibitors, 20 mM nethylmaleimide, 30 mM imidazole, and 25 U/mL Basemuncher (Expedeon). Protein concentrations of lysates were determined using Bradford assay. 1 μL of MultiDSK reagent (1 mg/mL) (Kerafast) was added to every 1,300 μL of lysate and incubated on a rotator for 1 hr at 4°C. NiNTA beads (Expedeon) were washed three times in lysis buffer (minimum 10× bead volume) before lysates were transferred and left overnight rotating at 4°C. Ni-NTA beads were washed three times in wash buffer (lysis buffer without nuclease) before bound complexes were eluted in 1× bead volume wash buffer containing 300 mM imidazole. Eluates were adjusted to 30 mM imidazole using lysis buffer; 2 μg anti-TDP1 antibody was added; and samples were rotated for 1 hr at 4°C. Protein A beads were washed three times in 10× bead volume wash buffer before the addition of Ni eluate, and samples were rotated at 4°C for 3 hr. Protein A beads were washed three times in wash buffer (20 mM Tris [pH 8], 150 mM NaCl, and 20 mM nethylmaleimide) before resuspension in 50 μL SDS loading buffer. Samples were then analyzed by immunoblotting using a 4%–12% gradient gel (BioRad TGX) and probed with anti-TDP1 antibody. Smear intensities were quantified using ImageJ. For *in vitro* deubiquitination assays, purified ubiquitylated TDP1 was incubated with 8.8 ng/μL BSA or recombinant UCHL3 (Thermo Fisher Scientific,11634H07E5) for 4 hr at 30°C in a deubiquitination buffer (50 mM Tris-HCL [pH 8.0], 50 mM NaCl,1 mM EDTA, 10 mM DTT, and 5% glycerol). Reaction products were analyzed by SDS-PAGE and immunoblotting. For detection of endogenous ubiquitylated TDP1, cells were lysed in 2% SDS, 150 mM NaCl, and 10 mM Tris-HCL (pH 8.0). Lysates were incubated at 90°C for 10 min, sonicated, and diluted 10× with dilution buffer (10 mM Tris-HCL [pH 8.0], 150 mM NaCl, 2 mM EDTA, and 1% Triton), followed by SDS-PAGE and immunoblotting.

### Alkaline Comet Assays

Alkaline comet assays (ACAs) were essentially conducted as previously described (Breslin et al., 2006). Briefly, HEK293T cells were transfected with control siRNA or UCHL3 siRNA, and 48 hr later, cells (∼3 × 10^5^ cells/sample) were suspended in media containing 14 μM CPT or DMSO for 20 min at 37°C. Approximately 5,000 cells were mixed with equal volumes of PBS and 1.2% type VII agarose at 42°C, plated on frosted microscope slides pre-coated with 0.6% agarose, and chilled until set. Cells were then lysed in 2.5 M NaCl, 10 mM Tris-HCl, 100 mM EDTA [pH 8], 1% Triton X-100, and 1% DMSO (pH 10) at 4°C for 1 hr and washed twice with cold distilled water. Slides were equilibrated in alkaline electrophoresis buffer (50 mM NaOH, 1 mM EDTA, and 1% DMSO) for 45 min and then subjected to electrophoresis at 12 V (100 mA) for 25 min. DNA was then stained by SYBR Green (1:2,000, Sigma S9430) for 5 min. Quantification of DNA breaks was performed using Comet Assay IV software and counting 150 cells per sample.

### Microarray Analyses

The dataset containing the microarray data from wild-type control and ataxia telangiectasia patient cerebella was downloaded from the Gene Expression Omnibus (https://www.ncbi.nlm.nih.gov/geo/) under the GEO accession GSE61019 (Jiang et al., 2015). The data were then normalized, and differential expression analysis was performed in R studio (R studio [v.1.0.136], R [v.3.3.2]) (Ritchie et al., 2015, Smyth, 2004). The microarray expression data were subjected to log2 normalization and presented as log2 mRNA levels.

### Experimental Repeats and Statistical Analyses

All data are presented as the means ± SEM of three experimental replicates, unless otherwise stated. Statistical differences were analyzed using the Student’s t test, with p < 0.05 considered to be statistically significant.

## Accession Numbers

The accession number for the raw data reported in this paper is Mendeley: 10.17632/rztbtg25zs.1. The accession number for the mass spectrometry data reported in this paper is Mendeley:10.17632/hp9vrpp56z.1.

## Supplemental Information

Document S1. Supplemental Experimental Procedures and Figures S1 and S2

Document S2. Article plus Supplemental Information
