# Supplementary material for: UCHL3 Regulates Topoisomerase-Induced Chromosomal Break Repair by Controlling TDP1 Proteostasis
Source: Cell Rep. 2018 Jun 13;23(11):3352–65. doi: 10.1016/j.celrep.2018.05.033 (PMC6019701; doi:10.1016/j.celrep.2018.05.033)
Supplement: Document S1. Supplemental Experimental Procedures and Figures S1 and S2 [file mmc1.pdf]

Cell Reports, Volume *23*

Supplemental Information

UCHL3 Regulates Topoisomerase-Induced Chromosomal Break Repair by Controlling TDP1 Proteostasis

Chunyan Liao, Ryan Beveridge, Jessica J.R. Hudson, Jacob D. Parker, Shih-Chieh Chiang, Swagat Ray, Mohamed E. Ashour, Ian Sudbery, Mark J. Dickman, and Sherif F. El-Khamisy

Supplementary Methods

Cell lines and Cell Culture

HEK293T and MRC5 cells were maintained as monolayers in MEM media supplemented with 10% FCS in a humidified atmosphere of 5% CO_2 at 37ºC. Wild-type AG87 and SCAN1 lymphoblastoid cell lines were maintained in suspension in RPMI media supplemented with 15% FCS and Penicillin/Streptomycin (Sigma). The human skeletal muscle cell-line (HSMM), obtained from Karl Morten, University of Oxford, was maintained in F10/Hams (Gibco,41550-021) with 10% FCS and Penicillin/Streptomycin. The rhabdomyosarcoma cell lines CW9019, a gift from Frederic G. Barr, NCI, Bethesda, was maintained in DMEM (Gibco 11965 with High Glucose and Glutamine, no pyruvate) with 10 % FCS and 1X Penicillin/Streptomycin.

Plasmids and antibodies

Expression plasmids encoding human TDP1 was described previously (Hudson et al., 2012). Flag-HA-UCHL3 vector was purchased from Addgene (Plasmid #22564). UCHL3 Was subcloned to pEGFP-N1 by Gibson Assembly Kit using primer pairs, Forward:CTCAAGCTTCGAATTCTGCAATGGAGGGTCAACG, Reverse: GGATCCCGGGCCCGCGGTACAGCTGCAGAAAGAGCAATCG. The UCHL3^{C95A} mutation was generated by site-directed mutagenesis using UCHL3^{C95A}F: GCAAACAATCAGCAATGCCGCAGGAACAATTGGACTGATTC and UCHL3^{C95A} R: GAATCAGTCCAATTGTTCCTGCGGCATTGCTGATTGTTTGC. Expression plasmids encoding HA-tagged ubiquitin mutants were gift from Simon Holst Bekker-Jensen. The antibodies used in this study are: anti-TDP1 (ab4166, Abcam), anti-Flag (M2, Sigma), anti-Myc (9E10, Santa Cruz), anti-HA (F-7, Santa Cruz), anti-UCHL3 (ab126703, Abcam), anti-tubulin (DM1A, Sigma), anti-β-actin (KM9001, Sungene Biotech), anti-53BP1 (A300-272A, Bethyl), anti-ubiquitin (linkage-specific K48) (Abcam, ab140601), anti-TOP1cc (Millipore, MABE1084), anti-GFP antibodies (ab290, Abcam), anti-mouse IgG HRP (85-18-8817-31, eBioscience), anti-rabbit IgG HRP (85-18-8816-31, eBioscience) and anti-USP11 (A301-613A, Bethyl).

CRISPR/Cas9 cell lines

For CRISPR/Cas9 knockout of human UCHL3 in HEK293T cells, the following small guide RNA sequence was used: 5'-CACCGTCGGAAGAGTCCAAGCGTGA-3', with the overhang sequence for BbsI restriction site. The gRNA sequences were cloned into the vector pSpCas9n(BB)-2A-puro. Cells were infected with pSpCas9-UCHL3-sgRNA-puro followed by extensive selection with 2 μg/mL puromycin, and single colonies were obtained by serial dilution and amplification. Clones were screened by immunoblotting with anti-UCHL3 antibody and verified by DNA sequencing.

Mass Spectroscopy

HEK293T cells were plated at a 5x10^{6} per 15 cm dish and transfected using a standard Calcium Phosphate precipitation. Plates were lysed in 1 ml lysis buffer (50 mM Tris pH8, 0.5% Triton X, 40 mM NaCl, 2 mM $CaCl_2$, 20 mM Nethylmaleimide, 1X protease inhibitor (Roche), 1X phosphatase inhibitor (Roche), 25 U/ml Basemuncher (Expedeon)). Immunoprecipitation was performed as described above. Samples were eluted in 100 μl elution buffer and separated by gel electrophoresis using a 4-12% gradient gel (BioRad TGX), followed by staining with the RAPID stain reagent (Gbiosciences) according to the manufacturer's instructions. Bands of interest were excised using a sterile scalpel and an in-gel trypsin digest performed as described (Pandey et al., 2000). Peptides were separated using an Ultimate 3000 liquid chromatography system (ThermoFisher, UK) and a 150 mm × 75 μm i.d. PepMap reversed phase column (ThermoFisher, UK). Linear gradient elution was performed from 95% buffer A (0.1% formic acid) to 50 % buffer B (0.1% formic acid, 95 % acetonitrile) at a flow rate of 300 ml/min in 60 mins. MS/MS analysis was performed using a maXis UHR TOF mass spectrometer (Bruker Daltonics) and a QExactive mass spectrometer (ThermoFisher) using data dependent acquisition. Spectra were deconvoluted and the peak lists exported as Mascot Generic Files (MGF) and searched using Mascot 2.2 server (Matrix Science). The Swissprot database was searched using a fixed FDR of 1% for peptide identifications. Tryptic enzyme specificity with up to two missed cleavages with oxidized methionine and GlyGly (K) used as a variable modification.

Quantification of protein turnover rates

2.5x10^{6} lymphoblastoid cells were resuspended in 3 ml media and 100μg/ml CHX were added to each sample 15 minutes prior to CPT treatment (final concentration 10μM CPT) for

the indicated time periods. HEK293T cells were seeded in 6 well plates, transfected with control siRNA or UCHL3 siRNA, after 48 hours, then incubated with CHX or CPT for the indicated time periods. Cells were harvested and analyzed by immunoblotting.

Quantitative real time PCR

Total RNA was isolated from 1×10^6 cells using RNeasy® Mini Kit (Qiagen, Valencia, CA). An aliquot of 1 µg RNA was reverse transcribed using a reverse transcription kit (Promega). Real-time PCR was performed with the SYBR® Green PCR Master Mix (Applied Biosystems, Foster city, CA) on the ABI 7900 thermocycler (Applied). Reaction mixtures contained 5 µl of 2X Quantitect SYBR-Green PCR Master Mix, 2 µl of reverse-transcriptase-generated cDNA diluted by 100 in a final volume of 10 µl containing primers (IDT) at 125 nM. Relative gene expression was expressed as a ratio of the expression level of the gene of interest to that of GAPDH, with values in control cells (AG87 or HSMM) defined as 100%. The sequences of TDP1 primers are Forward: CCCCTTCCAGTTTTACCTCAC, Reverse: AGTCCACGTCAAAGCAGTAG, GAPDH primers are Forward: ACATCGCTCAGACACCATG, Reverse TGTAGTTGAGGTCAATGAAGGG, UCHL3 primers are Forward: CTGAAGAACGAGCCAGATAC, Reverse: GCCCATCTACATGAACTAATGC

Immunofluorescence

MRC5 cells were plated on 13 mm round coverslips and incubated overnight. Cells were transfected with control siRNA or UCHL3 siRNA for 48 hours then treated with 1 µM CPT for 1 hour at 37°C. Cells were washed three times with PBS at the indicated time points and then fixed with 4 % paraformaldehyde for 15 minutes. Cold 0.2 % Triton was added for 2 minutes to permeate cell membranes; cells were then washed 3 times with PBS and incubated with 3 % BSA for 30 minutes. Cells were probed with anti-53BP1 antibodies for 60 minutes at room temperature, washed 3 times with PBS, then stained with anti-Alexa Fluor 555 antibody for 40 minutes and washed 3 times with PBS. The coverslips were transferred to 26 x 76 mm microscope slides and fixed with VectorShield mounting medium H-1000 (Vector). Cells were visualized on a Nikon E400 microscope and 53BP1 foci were quantified. Cells with more than 5 foci were considered positive. TOP1cc staining was essentially conducted as

described (Patel et al., 2016) with some modifications. HSMM and CW9019 cells grown on coverslips were treated with 1μM CPT or DMSO control for 10 min, fixed for 15 min at 4°C in 4% (w/v) paraformaldehyde in PBS, permeabilized with 0.25% (v/v) Triton X-100 in PBS for 2 min at 4°C. To render the DNA-protein crosslinks more accessible to antibody, the coverslips were incubated in 0.1% (w/v) SDS at 20-22°C for 5 min, washed five times with wash buffer [0.1% (w/v) bovine serum albumin and 0.1% (v/v) Triton X-100 in PBS. Blocked in TSM buffer consisting of 10% (w/v) powdered non-fat milk in 150 mM NaCl and 10 mM Tris-HCl (pH 7.4). After overnight incubation with the TOP1cc antibody (2 μg/ml) in PBS containing 5% (v/v) goat serum at 4°C, cells were washed 5 times with wash buffer, incubated with Alexa Fluor 488-conjugated secondary antibody at 1:1000 in PBS with 5% goat serum for 1 hour, washed 5 times with wash buffer and analysed by fluorescence microscopy.

Clonogenic survival assays

Cells transfected with control or UCHL3 siRNA were seeded at 500 – 2000 cells onto 10 cm petri dish and incubated overnight in complete media. Cells were incubated with the indicated concentrations of CPT or DMSO control for 1 hour at 37°C, washed twice with PBS, and then grown in drug-free media for 7 days. Cells were then fixed with 80 % ethanol for 15 minutes and stained with 1 % methylene blue for 1 hour. Surviving fraction was calculated by dividing the number of colonies on treated plates by the number of colonies on control plates. The average ± 1 standard error of the mean (SEM) was calculated from 3 independent biological repeats.

References

Hudson, J.J.R., Chiang, S.-C., Wells, O.S., Rookyard, C., El-Khamisy, S.F., 2012. SUMO modification of the neuroprotective protein TDP1 facilitates chromosomal single-strand break repair. Nature Communications 3, 733–13. doi:10.1038/ncomms1739

Pandey, A., Andersen, J.S., Mann, M., 2000. Use of mass spectrometry to study signaling pathways. Science's STKE 2000, pl1–pl1. doi:10.1126/stke.2000.37.pl1

Patel, A.G., Flatten, K.S., Peterson, K.L., Beito, T.G., Schneider, P.A., Perkins, A.L., Harki, D.A., Kaufmann, S.H., 2016. Immunodetection of human topoisomerase I-DNA covalent complexes. Nucleic Acids Res. 44, 2816–2826. doi:10.1093/nar/gkw109

Supplementary Figure 1

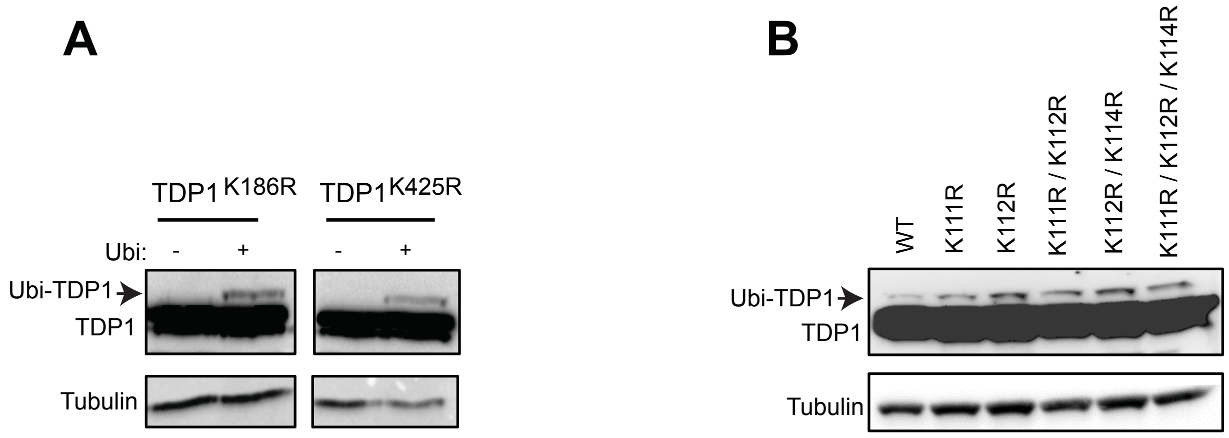

Suppl. Fig 1: Mutation of lysines 186, 425,111 or 112 to arginine did not abrogate TDP1 ubiquitylation (Related to Figure 1).(**A**) In silico analysis of TDP1 sequence using the ubiquitin site „ prediction tools UbPred, CKSAAP, and BDM-PUB revealed K186 and K425 as potential ubiquitylation sites. HEK293T cells were transfected with Myc-TDP ^{K186R} or Myc-TDP1^{K425R} and an empty plasmid “-y or a vector encoding His-Ubiquitin “+”. Lysates were fractionated on SDS-PAGE and blots analysed b Western blotting using with anti-TDP1 or anti-Tubulin antibodies. (**B**) Purified ubiquitylated TDP1 in Figure1D was subjected to mass spectrophotometric analysis using Amazon Ion Trap Maxis LC-QTOF or Thermo Orbitrap spectrometers. A low confidence potential site of modification was observed at lysine 114. Mutant variants of TDP1 were generated at K114 and the nearby lysine residue K112 in addition to the known SUMOylation site K111, either separately or together. HEK293T cells were transfected with Myc-TDP1 WT or indicated TDP1 mutants and a vector encoding His-Ubiquitin. Lysates were fractionated on SDS-PAGE and blots analyzed by Western blotting using with anti-TDP1 or anti-tubulin antibodies.

Supplementary Figure 2

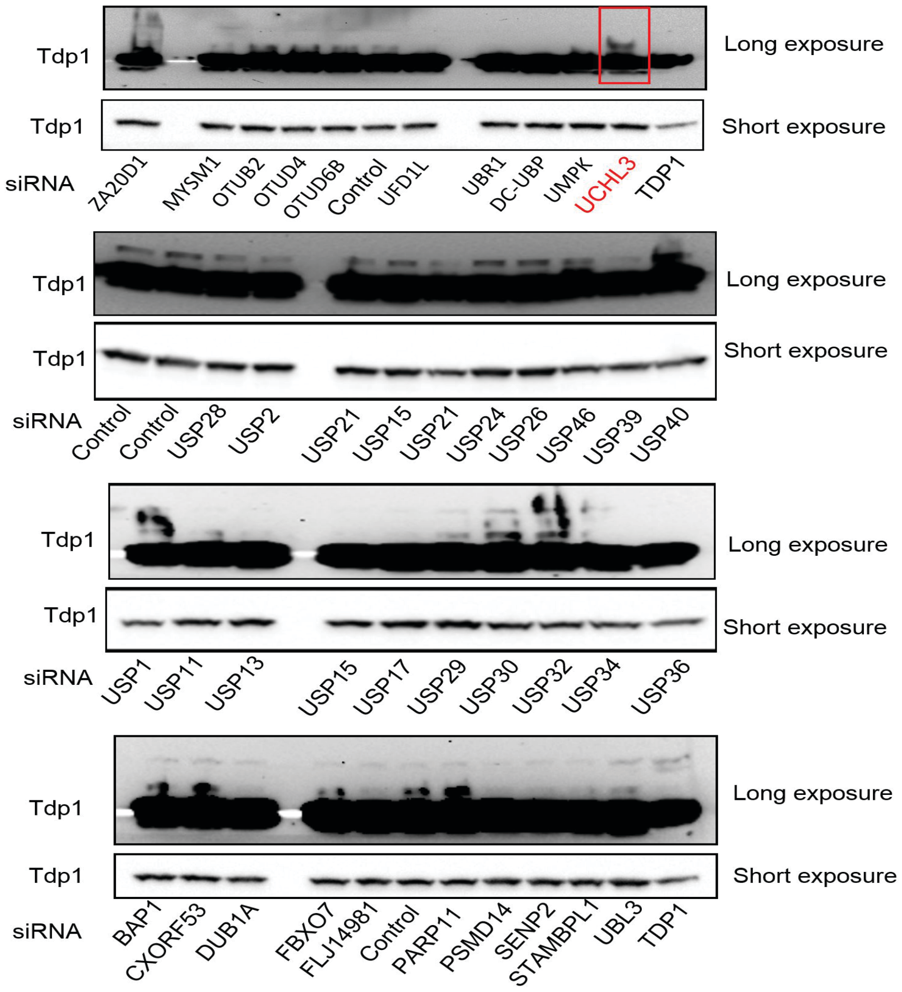

List of DUBs employed for the screen

BAP1	PARP11	USP1	USP29	USP47
COPS5	PRPF8	USP10	USP3	USP48
CXORF53	PSMD14	USP11	USP30	USP49
CYLD	SBBI54	USP12	USP31	USP5
DUB1A	SENP2	USP13	USP32	USP50
DUB3	STAMBP	USP14	USP33	USP51
FBXO7	STAMBPL1	USP15	USP34	USP52
FBXO8	TNFAIP3	USP16	USP35	USP53
FLJ14981	UBL3	USP17	USP36	USP54
JOSD1	UBL4	USP18	USP37	USP6
MJD	UBL5	USP19	USP38	USP7
MYSM1	UBR1	USP2	USP39	USP8
OTUB1	UBTD1	USP20	USP4	USP9X
OTUB2	DC-UBP	USP21	USP40	USP9Y
OTUD1	UCHL1	USP22	USP41	C13ORF22
OTUD4	UCHL3	USP24	USP42	VCPIP1
OTUD5	UCHL5	USP25	USP43	YOD1
OTUD6B	UMPK	USP26	USP44	ZA20D1
OTUD7	UEVLD	USP27X	USP45	ZRANB1
ZA20D1	UFD1L	USP28	USP46	

Suppl. Fig.2: siRNA screen to identify the DUB regulating TDP1 ubiquitylation (Related to Figure 1). A Human ON-TARGET plus siRNA library for all known DUBs (G-104705-05, GE Life Sciences) was re-plated from 96 to 24 well formats so that each would have a non-targeting control and TDP1 (transfection control) siRNA well. HEK293T cells were plated at $1x10^6$ per 10 cm dish and DNA transfected using a standard calcium phosphate precipitation. 24 hours after DNA transfection, siRNA library plates (24 well) were defrosted and 100 µl of MEM (not supplemented) containing 0.5 µl Dharmafect 1 (GE Life Sciences) added to each well and incubated for 20 minutes. DNA transfected cells were trypsinised, re-plated into the 24 well plates at a density of $2.2x10^5$/well and incubated for a further 48 hours. Cells were washed twice with 0.5 ml PBS, all PBS was removed before the addition of 40 µl SDS loading buffer, cells were vortexed 3 times for ~15 seconds. Lysates were then boiled for 10 minutes and clarified by centrifugation at 10,000g for 1 minute. Samples were analysed by immunoblotting using an 8% gel and anti TDP1 antibody. The screen was repeated 4 times and a representative replica is shown. UCHL3 was a consistent hit across the 4 screens.
